# Characterization of *Mycobacterium chelonae*-Like Strains by Comparative Genomics

**DOI:** 10.3389/fmicb.2017.00789

**Published:** 2017-05-08

**Authors:** Christiane L. Nogueira, Luiz G. P. de Almeida, Maria C. Menendez, Maria J. Garcia, Luciano A. Digiampietri, Erica Chimara, Margo Cnockaert, Juan C. Palomino, Françoise Portaels, Anandi Martin, Peter Vandamme, Sylvia C. Leão

**Affiliations:** ^1^Departamento de Microbiologia, Imunologia e Parasitologia, Escola Paulista de Medicina, Universidade Federal de São PauloSão Paulo, Brazil; ^2^Laboratório Nacional de Computação CientíficaPetrópolis, Brazil; ^3^Departamento de Medicina Preventiva, Facultad de Medicina, Universidad Autónoma de MadridMadrid, Spain; ^4^Escola de Artes, Ciências e Humanidades, Universidade de São PauloSão Paulo, Brazil; ^5^Núcleo de Tuberculose e Micobacterioses, Instituto Adolfo LutzSão Paulo, Brazil; ^6^Laboratory of Microbiology, Faculty of Sciences, Ghent UniversityGhent, Belgium; ^7^Mycobacteriology Unit, Department of Biomedical Sciences, Institute of Tropical MedicineAntwerpen, Belgium

**Keywords:** mycobacterium, *M. chelonae*-*M. abscessus* complex, whole genome sequencing, taxonomy, identification

## Abstract

Isolates of the *Mycobacterium chelonae*-*M. abscessus* complex are subdivided into four clusters (CHI to CHIV) in the INNO-LiPA® *Mycobacterium* spp DNA strip assay. A considerable phenotypic variability was observed among isolates of the CHII cluster. In this study, we examined the diversity of 26 CHII cluster isolates by phenotypic analysis, drug susceptibility testing, whole genome sequencing and single-gene analysis. Pairwise genome comparisons were performed using several approaches, including average nucleotide identity (ANI) and genome-to-genome distance (GGD) among others. Based on ANI and GGD the isolates were identified as *M. chelonae* (14 isolates), *M. franklinii* (2 isolates) and *M. salmoniphium* (1 isolate). The remaining 9 isolates were subdivided into three novel putative genomospecies. Phenotypic analyses including drug susceptibility testing, as well as whole genome comparison by TETRA and delta differences, were not helpful in separating the groups revealed by ANI and GGD. The analysis of standard four conserved genomic regions showed that *rpoB* alone and the concatenated sequences clearly distinguished the taxonomic groups delimited by whole genome analyses. In conclusion, the CHII INNO-LiPa is not a homogeneous cluster; on the contrary, it is composed of closely related different species belonging to the *M. chelonae-M. abscessus* complex and also several unidentified isolates. The detection of these isolates, putatively novel species, indicates a wider inner variability than the presently known in this complex.

## Introduction

The *Mycobacterium chelonae*-*M. abscessus* complex consists of closely related rapidly growing mycobacteria. According to the classification proposed by Runyon (1965), rapid growing mycobacteria include species that produce visible colonies on solid medium in <7 days. Although ubiquitous environmental organisms, they can cause several opportunistic infections in humans, especially pulmonary and skin infections (Wallace et al., 1983; Brown-Elliott and Wallace, 2002; Whipps et al., 2007). This complex is the most commonly identified mycobacterial group causing diseases in humans after the *Mycobacterium tuberculosis* and *Mycobacterium avium* complexes (Sassi and Drancourt, 2014). Nowadays, *M. abscessus* is one of the main infectious agents causing respiratory exacerbation in patients with cystic fibrosis (Bryant et al., 2013).

Several changes in the classification of the members of the *M. chelonae*-*M. abscessus* complex have occurred over the years. Currently the species that are formally accepted include *M. chelonae, M. abscessus* (Kusunoki and Ezaki, 1992)—with three subspecies, *M. abscessus* subsp. *abscessus, M. abscessus* subsp. *massiliense*, and *M. abscessus* subsp. *bolletii* (Leao et al., 2009, 2011; Tortoli et al., 2016), *M. immunogenum* (Wilson et al., 2001), *M. salmoniphilum* (Ross, 1960; Whipps et al., 2007), *M. franklinii* (Simmon et al., 2011; Nogueira et al., 2015a), and *M. saopaulense* (Nogueira et al., 2015b).

Despite technological advances, accurate species level identification of *M. chelonae*-*M. abscessus* complex bacteria represents a challenge for clinical laboratories. In general, these species have very similar phenotypic characteristics (Simmon et al., 2011; Nogueira et al., 2015a,b). Moreover, partial 16S rDNA sequences are too similar, underestimating their diversity and not distinguishing all taxa (Adékambi et al., 2003; Simmon et al., 2011). *M. chelonae-M. abscessus* complex members can be differentiated by the analysis of DNA polymorphisms in the *rpoB* and *hsp65* genes and in the 16S–23S rRNA internal transcribed spacer (ITS-1). However, Adékambi et al. (2003) demonstrated that *M. abscessus* isolates have >4.3% *rpoB* sequence divergence, which is a considerable intra species variability that adds another challenge to the identification of *M. chelonae-M. abscessus* complex bacteria.

A considerable variability was also observed among *M. chelonae* isolates during the development of a DNA strip assay named INNO-LiPA® *Mycobacterium* spp (Innogenetics, Belgium). This reverse hybridization line probe assay was developed based on the high ITS-1 sequence heterogeneity of mycobacteria. DNA probes specific for the clinically important mycobacterial species were selected, including a set of 9 probes specific for the *M. chelonae-M. abscessus* complex that allowed the subdivision of isolates from this group into four clusters (CHI, CHII, CHIII, and CHIV) according to their hybridization profiles (Portaels et al., 1996). The commercial version of this test used only 3 probes, MCH-1, MCH-2, and MCH-3. Isolates that showed hybridization with probes MCH-1 and MCH-3 were identified as cluster CHI. Cluster CHIII showed hybridization with probes MCH-1 and MCH-2, and clusters CHII and CHIV only with probe MCH-1. *M. abscessus* isolates and the type strain ATCC 19977^T^ were encompassed in the CHIII cluster (Portaels et al., 1996). Interestingly, variability in phenotypic characteristics of isolates belonging to CHII cluster was observed, suggesting the existence of different taxonomic entities within the group. Previous publications indicated that *M. chelonae* isolates cannot grow in the presence of 5% NaCl and can use citrate as the sole carbon source while *M. abscessus* is tolerant to 5% NaCl and can utilize sodium citrate as the sole carbon source (Leao et al., 2004). However, some CHII isolates showed conflicting results by these tests (Portaels et al., 1996).

To explore the variability observed during the development of INNO-LiPA assay, a set of CHII cluster isolates was studied. Whole genome sequencing and pairwise genome comparisons were performed to better understand the diversity of the CHII cluster. The ability of DNA targets commonly used for identification of mycobacteria in discriminating the groups separated by genomic comparisons was also verified.

## Materials and methods

### Isolates, reference strains and growth media

This study was carried out with 26 isolates belonging to INNO-LiPa cluster CHII recovered from clinical and environmental specimens by Prof. Françoise Portaels (Institute of Tropical Medicine, Antwerp, Belgium) and Prof. Roland Schulze-Röbbecke (University of Dusseldorf, Dusseldorf, Germany). *M. smegmatis* mc^2^155, *M. tuberculosis* H37Rv and the type strains of *M. chelonae-M. abscessus* complex (*M. abscessus* subsp. *abscessus* ATCC 19977^T^, *M. abscessus* subsp. *bolletii* CCUG 50184^T^, *M. abscessus* subsp. *massiliense* CCUG 48898^T^, *M. chelonae* ATCC 35752^T^, *M. immunogenum* ATCC 700505^T^, *M. salmoniphilum* ATCC 13758^T^, *M. franklinii* DSM 45524^T^, and *M. saopaulense* CCUG 66554^T^) were included for comparison (Table 1).

**Table 1:** **Isolates and type strains of *M. chelonae*-*M. abscessus* complex included in this study**.

**Isolate**	**Isolation Source**	**Procedence**	**INNO-LiPA**
96-1705	Human foot biopsy	ITM, Belgium	CHII
96-1717	Human hand tissue	ITM, Belgium	CHII
96-1720	Human leg abscess	ITM, Belgium	CHII
96-1724	Human leg abscess	ITM, Belgium	CHII
96-1728	Lizard liver tissue	ITM, Belgium	CHII
D16R27	Tap water	UD, Germany	CHII
D16Q13	Tap water	UD, Germany	CHII
D16Q14	Tap water	UD, Germany	CHII
D16Q15	Tap water	UD, Germany	CHII
D16Q24	Tap water	UD, Germany	CHII
D16R2	Tap water	UD, Germany	CHII
D16R3	Tap water	UD, Germany	CHII
D16R7	Tap water	UD, Germany	CHII
D16R9	Tap water	UD, Germany	CHII
D16R14	Tap water	UD, Germany	CHII
D16R18	Tap water	UD, Germany	CHII
D16R19	Surface water	UD, Germany	CHII
D16R20	Surface water	UD, Germany	CHII
D16R10	Surface water	UD, Germany	CHII
D16R12	Surface water	UD, Germany	CHII
D17A2	Water work	UD, Germany	CHII
D16Q19	Water work	UD, Germany	CHII
D16Q16	Water work	UD, Germany	CHII
D16Q20	Water work	UD, Germany	CHII
D16R24	Water work	UD, Germany	CHII
96-892	–	ITM, Belgium	CHII
*M. abscessus* subsp. *abscessus*	–	ATCC 19977^T^	CHIII
*M. abscessus* subsp. *bolletii*	–	CCUG 50184^T^	ND
*M. abscessus* subsp. *massiliense*	–	CCUG 48898^T^	ND
*M. chelonae*	–	ATCC 35752^T^	CHII/IV
*M. immunogenum*	–	ATCC 700505^T^	ND
*M. salmoniphilum*	–	ATCC 13758^T^	ND
*M. franklinii*	–	DSM 45524^T^	ND
*M. saopaulense*	–	CCUG 66554^T^	ND

*UD, University of Dusseldorf, Dusseldorf, Germany; ITM, Institute of Tropical Medicine Prince Leopold, Antwerp, Belgium; ND, not determined*.

Cultures were grown aerobically at 28–30°C on solid media including Löwenstein-Jensen (LJ) and Middlebrook 7H10 [Becton-Dickinson (BD), USA] supplemented with oleic acid, albumin, dextrose and catalase (OADC–BD) and in liquid media including Middlebrook 7H9 (BD), Mueller-Hinton and Lisogeny Broth with 1% Tween 80.

### Phenotypic analyses

Phenotypic analyses were performed as described in standard protocols for biochemical identification of mycobacteria (Tsukamura, 1984; Kent and Kubica, 1985; Leao et al., 2004). Analysis of pigment production, single-source carbon utilization (mannitol, inositol and citrate), growth at 26° and 37°C and tolerance to 5% NaCl, 0.2% picric acid, 0.2% nitrite and para-nitrobenzoic acid (PNB) were performed on 7H10-OADC and LJ. Nitrate reduction, Tween 80 hydrolysis and arylsulfatase production were also examined.

### Susceptibility testing

Antimicrobial drug-susceptibility testing was performed using the microdilution method in cation-supplemented Mueller–Hinton broth, according to the recommendations of the Clinical and Laboratory Standards Institute [Clinical and Laboratory Standards Institute (CLSI), 2011] for rapidly growing mycobacteria. The antimicrobials tested were amikacin, cefoxitin, ciprofloxacin, clarithromycin, doxycycline, minocycline, moxifloxacin and tobramycin.

### DNA extraction

Chromosomal DNA was extracted using QIAamp DNA mini kit (Qiagen, Germany) as previously described (Bryant et al., 2013). DNA concentration was determined using a Qubit high-sensitivity (HS) assay kit (Life Technologies, USA).

### Whole genome sequencing and assembly

High quality DNA of the 26 isolates and of *M. abscessus* subsp. *bolletii* CCUG 50184^T^, *M. immunogenum* ATCC 700505^T^, *M. salmoniphilum* ATCC 13758^T^, and *M. franklinii* DSM 45524^T^ were subjected to multiplexed paired end sequencing using the Illumina Miseq platform. The genome of *M. saopaulense* CCUG 66554^T^ was sequenced in a previous project from the laboratory of the Universidade Federal de São Paulo (accession number CP010271). The genomes of *M. abscessus* subsp. *abscessus* ATCC19977^T^ (accession number CU458896), *M. abscessus* subsp. *massiliense* CCUG48898^T^ (accession number NZ_AKVF01000005NZ_AKVF01000001 to NZ_AKVF01000005), and *M. chelonae* ATCC 35752^T^ (accession number CP010946) were retrieved from the GenBank database (http://www.ncbi.nlm.nih.gov/genbank/). Sequencing errors in reads were corrected with the program Quake v0.3 (Kelley et al., 2010) and reads trimmed with the program Trimmomatic v0.33 (Bolger et al., 2014). The assembly was performed with Newbler program v3.0 (20140318_1550)—version with support for reads with Illumina's Casava accession number v1.8 format) using default parameters. Raw sequencing data was deposited on the NCBI Sequence Read Archive (http://www.ncbi.nlm.nih.gov/sra) under accession number SRP075879 and the assembled genomes were deposited as BioProject PRJNA323571.

### Procedures of whole genome sequence comparison

#### Average nucleotide identity (ANI) and tetranucleotide frequency correlation coefficients (TETRA) analysis

ANI by BLAST (ANIb) and by MUMmer (ANIm) and TETRA-nucleotide usage patterns were calculated using JSpecies v1.2.1. Cutoff values for species separation were <95% ANIb and ANIm and <0.99 TETRA (Kurtz et al., 2004; Teeling et al., 2004; Goris et al., 2007). A tree based on the obtained ANIb values was constructed using MEGA7 software (Saitou and Nei, 1987; Kumar et al., 2016) using the genomes of *M. smegmatis* mc^2^155 (accession number NC_018289) and *M*. *tuberculosis* H37Rv (accession number NC_018143) as outgroups.

#### Genome-to-genome distance (GGD) calculations

GGD was calculated using the Genome-to-Genome Distance Calculator (GGDC at http://ggdc.dsmz.de). The distance values between the genomes were determined and the digital DNA-DNA hybridization (dDDH) was calculated from these distances. Cutoff values for species discrimination were ≥0.0258 distance value and <70% dDDH (Meier-Kolthoff et al., 2013). A tree based on GGD values was constructed using MEGA7 software (Saitou and Nei, 1987; Kumar et al., 2016) using the genomes of *M. smegmatis* mc^2^155 (accession number NC_018289) and *M*. *tuberculosis* H37Rv (accession number NC_018143) as outgroups.

#### Genomic signature (delta values)

The relative abundance of di-, tri- and tetra-nucleotides distributed along the genomes was calculated using the program available at http://www.cmbl.uga.edu/software/delta-differences.html. The delta value obtained by comparing a genome with itself is considered the threshold for species separation for that particular genome (Karlin et al., 1997).

### Comparison of isolates by single-gene sequencing

Taxonomically informative partial sequences of 16S rDNA, *rpoB, hsp65* and 16S–23S ITS-1 fragments were PCR amplified and sequenced using primers listed in Supplementary Table 1. PCR products were purified using QIAquick PCR purification Kit (Qiagen, Germany). Dideoxy sequencing was performed using BigDye® 19 Terminator v3.1 Cycle Sequencing Kit (Applied Biosystems, USA) and run in ABI PRISM 3100 DNA Analyzer (Applied Biosystems).

Individual and concatenated phylogenetic trees based on the partial sequences of the previous genomic regions were constructed using PhyML (http://www.atgc-montpellier.fr/phyml/) (Guindon and Gascuel, 2003), using as input the multiple alignment of these sequences produced by MUSCLE (http://www.ebi.ac.uk/Tools/msa/muscle/help/) with default parameters (penalty for gap opening = 400, gap extension = 0). Confidence bootstrap values were calculated with 100 replicates. The corresponding sequences of the *M. chelonae*-*M. abscessus* complex type strains and outgroups (*M. tuberculosis* H37Rv and *M. smegmatis* mc^2^155) were retrieved from the GenBank database (http://www.ncbi.nlm.nih.gov/genbank/) (Supplementary Table 2).

### GenBank/EMBL/DDBJ accession numbers

The 16S rDNA, *hsp65*, 16S–23S ITS-1 and *rpoB*, partial sequences obtained in this study were deposited in the GenBank/EMBL/DDBJ under accession numbers: KT779789, KT779792-KT779795, and KT779797-KT779815 (16S rDNA), KT779818, KT779821-KT779824 and KT779826-KT779844 (*hsp65*), KT779847, KT779850-KT779853, and KT779855-KT779873 (16S–23S ITS-1), and KT779876, KT779879-KT779882, and KT 779884-KT779902 (*rpoB*).

The genomes were deposited in the GenBank/EMBL/DDBJ under accession numbers: MAEQ00000000 (*M. chelonae* 96-1705), MAER00000000 (*M. chelonae* 96-1717), MAES00000000 (*M. chelonae* 96-1720), MAET00000000 (*M. chelonae* 96-1724), MAEU00000000 (*M. chelonae* 96-1728), MAEV00000000 (*M*. sp. D16R24), MAEP00000000 (*M. franklinii* D16R27), MAEW00000000 (*M*. sp. D16Q13), MAEX00000000 (*M*. sp. D16Q14), MAEY00000000 (*M*. sp. D16Q16), MAFS00000000 (*M. franklinii* D16Q19), MAEZ00000000 (*M*. sp. D16Q20), MAFA00000000 (*M. chelonae* D16Q24), MAFB00000000 (*M*. sp. D17A2), MAFC00000000 (*M*. sp. D16R12), MAFD00000000 (*M*. sp. D16R18), MAFE00000000 (*M. salmoniphilum* D16Q15), MAFF00000000 (*M. chelonae* D16R2), MAFG00000000 (*M. chelonae* D16R3), MAFH00000000 (*M. chelonae* D16R7), MAFI00000000 (*M. chelonae* D16R9), MAFJ00000000 (*M. chelonae* D16R10), MAFK00000000 (*M. chelonae* D16R14), MAFL00000000 (*M. chelonae* D16R19), MAFM00000000 (*M. chelonae* D16R20), MAFN00000000 (*M*. sp. 96-892), MAFO00000000 (*M. abscessus* subsp. *bolletii* BD), MAFP00000000 (*M. immunogenum* MC 779), MAFQ00000000 (*M. franklinii* CV002), and MAFR00000000 (*M. salmoniphilum* SC).

## Results

### Phenotypic analyses and drug susceptibility testing

The type strains of the eight formally named members of the *M. chelonae-M. abscessus* complex and 26 isolates from the INNO-LiPA cluster CHII were analyzed. Five isolates from clinical specimens (96-1705, 96-1717, 96-1720, 96-1724, and 96-892) and one from an animal (96-1728) were received from the Institute of Tropical Medicine in Antwerp, Belgium. The remaining 20 isolates were obtained from water sources in Germany and were received from the collection of the University of Dusseldorf in Dusseldorf, Germany.

All CHII isolates and type strains grew in the presence of picric acid, 5% NaCl at 30°C and PNB and generated nonchromogenic colonies on solid culture media within 7 days. They did not reduce nitrate or hydrolyze Tween 80, but exhibited arylsulfatase activity within 3 days.

Growth in the presence of nitrite and 5% NaCl at 37°C and utilization of mannitol, inositol and citrate as single-source carbon sources, generated strain specific results and were not consistently related to any of the established species within this complex (Supplementary Table 3).

All isolates tested were susceptible to clarithromycin and resistant to cefoxitin, except for *M. franklinii* DSM 45524^T^, which was susceptible to cefoxitin (MIC = 16 μg/mL). Variable results were obtained with the other tested antimicrobials (Supplementary Table 4).

### Whole genome sequencing and assembly

The number of assembled bases ranged from 4,768,278 to 5,548,818, with an average G+C content of 63.94%. The number of generated scaffolds ranged from 15 to 82 with an N50 size from 110,426 to 682,599 bp. Genome sizes were consistent with the expected sizes of known species within the complex (Supplementary Table 5).

### Average nucleotide identity (ANI)

Average Nucleotide Identity (ANI) compares the nucleotide sequences of conserved genes shared by two genomes. ANI comparison measures the level of identity of nucleotides after full alignment of two genomes and selection of the most conserved regions, in such a way that only highly conserved genes are compared. This characteristic made ANI very popular in whole genome sequencing (WGS) comparative studies, because it is considered to represent more accurately the evolutionary relationships among genomes.

Pairwise ANIb and ANIm values of the *M. chelonae-M. abscessus* complex type strains were all below 95%, except between *M. abscessus* subsp. *abscessus* ATCC 19977^T^, *M. abscessus* subsp. *bolletii* CCUG 50184^T^ and *M. abscessus* subsp. *massiliense* CCUG 48898^T^. These results confirmed that the type strains represent distinct species within the *M. chelonae-M. abscessus* complex and that the strains ATCC 19977^T^, CCUG 50184^T^, and CCUG 48898^T^ are appropriately classified into a single species, *M. abscessus* (Table 2 and Supplementary Table 6).

**Table 2:** **Taxonomic groups based on results of ANI, GGD, dDDH, TETRA, and delta differences**.

**Isolates**	**ANI**		**GGD**		**dDDH**		**TETRA**		**delta differences**	
	**A**	**B**	**A**	**B**	**A**	**B**	**A**	**B**	**A**	**B**
96–1705	>95%	>95% ATCC 35752^T^	<0.0258	>0.0258	>70%	<70%	>0.99	>0.99	21–27	21–27
96–1717										
96–1720										
96–1724										
96–1728										
D16Q24										
D16R2										
D16R3										
D16R7										
D16R9										
D16R10										
D16R14										
D16R19										
D16R20										
D16Q15	–	>95% ATCC 13758^T^	–	>0.0258	–	>70% ATCC 13758^T^	>0.99	>0.99	21–27	21–27
D16Q19	>95%	>95% DSM 45524^T^	<0.0258	>0.0258	>70%	>70% DSM 45524^T^	>0.99	>0.99	21–27	21–27
D16R27										
D16Q14	>95%	<95%	<0.0258	>0.0258	>70%	<70%	>0.99	>0.99	21–27	21–27
D16Q20										
D16R24										
D17A2										
96-892	>95%	<95%	<0.0258	>0.0258	>70%	<70%	>0.99	>0.99	21–27	21–27
D16Q16										
D16Q13										
D16R12	>95%	<95%	>0.0258	>0.0258	<70%	<70%	>0.99	>0.99	21–27	21–27
D16R18										

*(A) results between isolates inside each group and (B) results between each group of isolates and the M. chelonae-M. abscessus type strains*.

**Figure 1:**
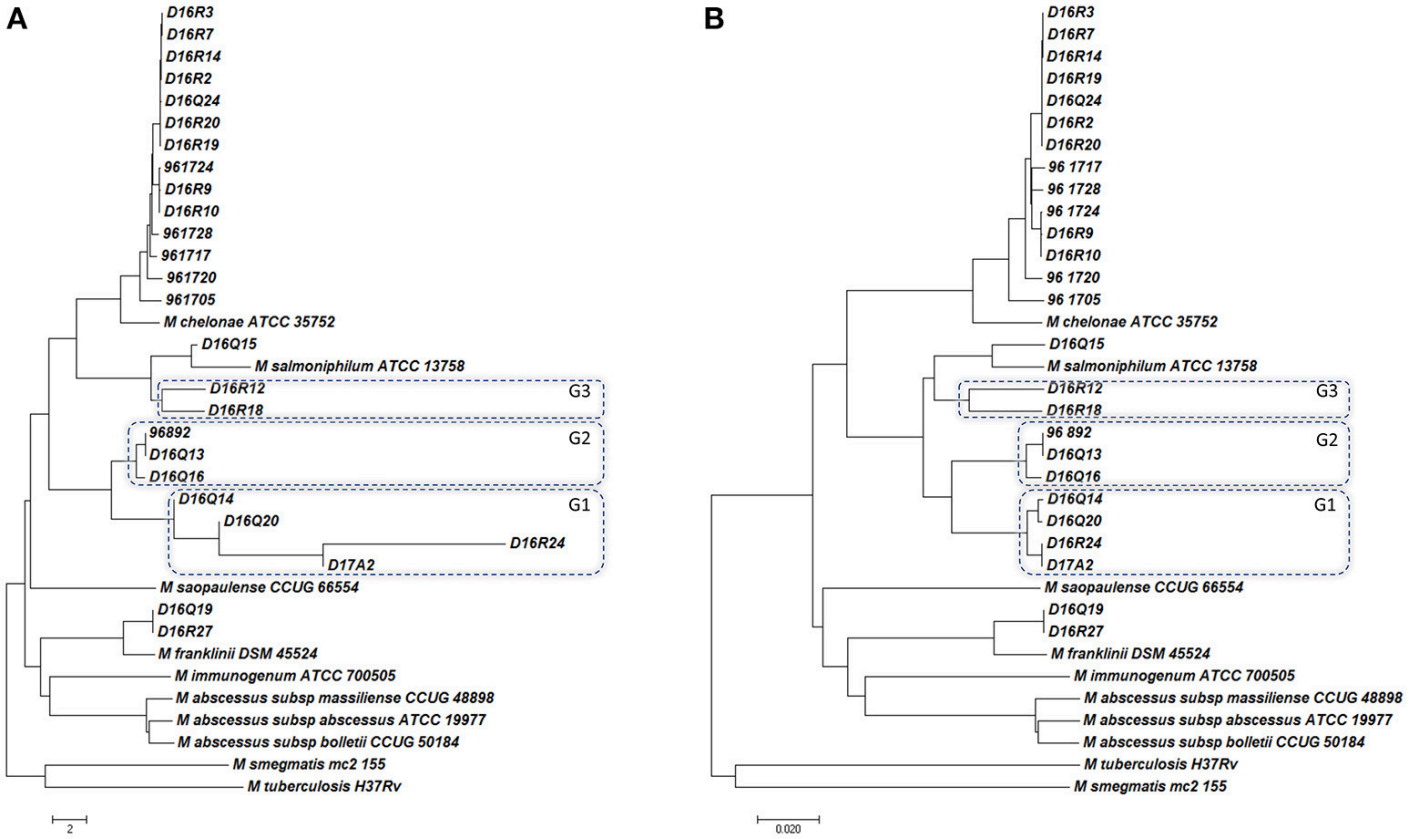
**The evolutionary history was inferred using the Neighbor-Joining method (Saitou and Nei, 1987)**. The tree is drawn to scale, with branch lengths in the same units as those of the evolutionary distances used to infer the phylogenetic tree. Evolutionary analyses were carried out in MEGA7 (Kumar et al., 2016). **(A)** Tree based on ANIb analysis; **(B)** Tree based on GGD analysis. The novel genomospecies G1, G2, and G3 are highlighted in boxes.

Based on ANIb and ANIm values, the CHII isolates could be separated into different taxonomic groups. The pairwise genome alignment of 14 isolates (96-1705, 96-1717, 96-1720, 96-1724, 96-1728, D16Q24, D16R2, D16R3, D16R7, D16R9, D16R10, D16R14, D16R19, and D16R20) yielded >95% ANIb and ANIm values, showing that they belong to the same species. The ANI values between these isolates and the type strains yielded values slightly higher than 95% with *M. chelonae* ATCC 35752^T^, showing that they could be classified into the species *M. chelonae* according to their ANI. Isolate D16Q15 yielded ANIb and ANIm values above 95% with *M. salmoniphilum* ATCC 13758^T^, showing that it belongs to the species *M. salmoniphilum* by this approach; and isolates D16Q19 and D16R27 yielded ANIb and ANIm values above 95% with *M. franklinii* DSM 45524^T^, indicating that they belong to the species *M. franklinii*, thus confirming previously reported data (Nogueira et al., 2015a). The remaining nine isolates yielded ANI values below 95% with all type strains indicating a clear separation from the *M. chelonae-M. abscessus* complex at the species level. These isolates could be grouped in three genomospecies using their ANI values: D16Q14, D16Q20, D16R24, and D17A2 (Genomospecies G1), 96-892, D16Q13, and D16Q16 (Genomospecies G2); and D16R12 and D16R18 (Genomospecies G3). Pairwise ANI values of isolates within each genomospecies were above 95% (Table 2 and Supplementary Table 6). The complete ANIb data distribution was represented in a tree (Figure 1A). The tree obtained using ANIm data showed the same distribution (data not shown).

### Tetranucleotide frequency correlation coefficients (TETRA) analysis

Tetranucleotide frequency correlation coefficients (TETRA) analysis determines the relative tetra-oligonucleotide invariance along the genome sequence, including coding- and non-coding regions. The procedure is based on the hypothesis that the composition of tetra nucleotide sequences in a genome is conserved within a species; moreover, the level of similarity of that composition is related to the evolutionary distance between genomes. Two highly similar genomes would have higher than 99% TETRA coefficient (Teeling et al., 2004). Values above this percentage mean that the bacteria belong to the same species.

The pairwise TETRA coefficients were all above the 0.99 threshold, even between the different type strains (Table 2). Isolates with pairwise ANI values above 95% showed TETRA values above 0.999 and pairwise ANI values below 95% corresponded to TETRA values between 0.99 and 0.999. ANI values below 95% and TETRA values above 0.999 were obtained in pairwise comparisons of D16Q16 and D16Q20, D16R18 and D16Q15, D16R18 and *M. salmoniphilum* ATCC 13758^T^ (Supplementary Table 7). These results showed a low discriminative power of TETRA analysis applied to the CHII group.

### Genome-to-genome distance (GGD) calculations

Genome-to-Genome Distance (GGD) calculation is a web-based procedure that performs *in silico* genome-to-genome comparison. The method is based on BLAST nucleotide comparison of entire sequences and allows calculation of digital DNA-DNA hybridization (dDDH) values, corresponding to classical wet-lab DDH.

The genome distance displayed by the genomes under study confirmed the grouping obtained with ANI, however some partially discordant data were observed (Table 2, Figure 1B and Supplementary Table 8). The 14 isolates found to belong to *M. chelonae* by ANI, showed data suggesting that they could belong to a different species very closely related to *M. chelonae* ATCC 35752^T^ (GGD around 0.045 and dDDH around 64%) (Supplementary Table 8). A similar result was found when comparing genomospecies G3 genomes to each other (isolates D16R12 and D16R18) (Supplementary Table 8), while GGD and dDDH data confirmed data for genomospecies G1 and G2 (Table 2).

On the other hand, isolate D16Q15 vs. *M. salmoniphilum* ATCC 13758^T^ showed GGD values slightly higher than the accepted threshold (0.0340, see Supplementary Table 8). This result could suggest that they belong to different species; yet, dDDH percentage values higher than 70% were obtained, confirming that they should be considered as a single species. The same situation was seen when comparing D16Q19 and D16R27 vs. *M. franklinii* DSM 45524^T^, with GGD value of 0.0336 and 0.0335, respectively and calculated dDDH of 71.90% (Table 2).

As expected, the genomes from reference type-strains showed GGD and dDDH values corresponding to those of different species (GGD between 0.1272 and 0.1627; dDDH <33%; Supplementary Table 8). When comparing genomes of *M. abscessus* subspecies to each other, genome distance analysis showed values slightly higher than the threshold (0.0266–0.0288) corresponding to dDDH >70%, (Supplementary Table 8).

Data obtained of the genome distance, represented in a tree (Figure 1B), showed similar genome distribution to that derived from ANI data (Figure 1A).

### Genomic signature (delta values)

Genomic Signature determines the relative intragenomic invariance of di- or tetra-oligonucleotide composition along the genome sequence, similarly to TETRA analysis. Similarities among genomes are represented as delta asterisk (δ^*^) values (see Supplementary Table 9). There is no general threshold for species separation using this approach. The calculated δ^*^ value, when a genome is compared with itself, represents the threshold value that is used for species separation for the considered genome. Higher values identify genomes of different species and equal to or lower values identify genomes of the same species.

The obtained delta values fell within the range of the calculated cutoff values, between 21 and 27, indicating that all isolates and type strains are closely related. The type strains *M. abscessus* subsp. *abscessus* ATCC 19977^T^, *M. abscessus* subsp. *bolletii* CCUG 50184^T^ and *M. abscessus* subsp. *massiliense* CCUG 48898^T^ showed the lowest delta values (22 to 24) and, as expected, were grouped as a single species. In accordance with the distribution found with ANI and GGD, the three *M. abscessus* subspecies and *M. immunogenum* ATCC 700505^T^ appeared more separated from the other strains and isolates within the group (delta values of 24 to 28) (Table 2 and Supplementary Table S9).

### Single-gene analyses

**Figure 2:**
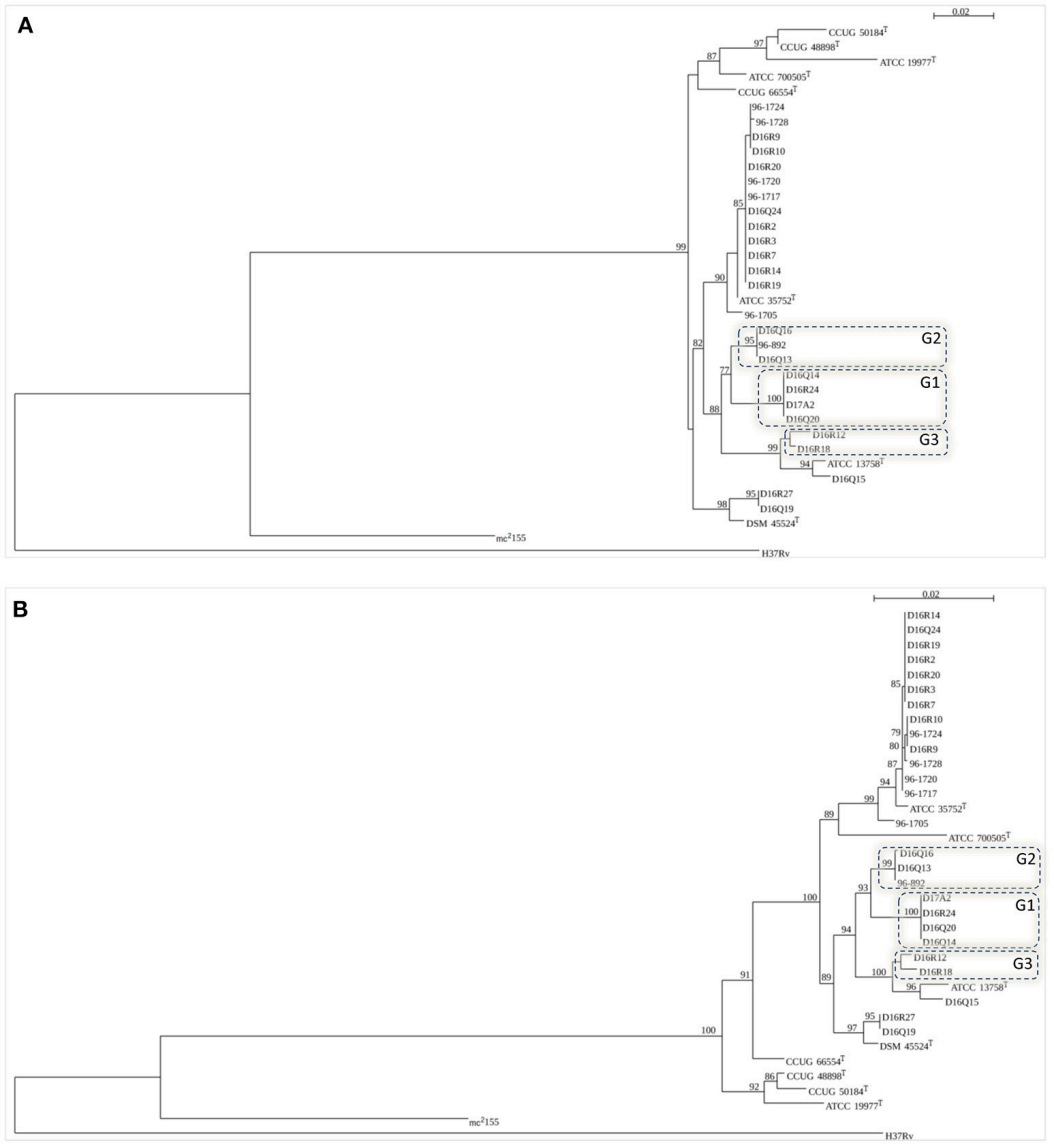
**Trees based on the figure generated by SeaView (http://doua.prabi.fr/software/seaview)**. Bootstrap values >50% are shown at nodes. **(A)** *rpoB* (711 bp); **(B)** concatenated sequences of 16S rDNA (1384 bp), *hsp65* (401 bp), 16S–23S ITS fragment (214 bp) and *rpoB* (711 bp). Type strains included in the trees: *M. abscessus* subsp. *abscessus* (ATCC 19977^T^), *M. abscessus* subsp. *bolletii* (CCUG 50184^T^) *M. abscessus* subsp. *massiliense* (CCUG 48898^T^), *M. chelonae* (ATCC 35752^T^), *M. immunogenum* (ATCC 700505^T^), *M. franklinii* (DSM 45524^T^), *M. salmoniphilum* (ATCC 13758^T^), and *M. saopaulense* (CCUG 66554^T^). *M. tuberculosis* H37Rv and *M. smegmatis* mc^2^155 were used as outgroups. The novel genomospecies G1, G2, and G3 are included in boxes.

Individual trees obtained with 16S rDNA, *rpoB, hsp65*, and ITS-1 sequences grouped the CHII isolates among members of *M. chelonae*-*M. abscessus* complex (Figure 2 and Supplementary Figure 1).

The individual trees obtained with 16S rDNA, *hsp65*, and ITS sequences showed some discordant groupings when compared to ANI and GGD trees (Supplementary Figure 1). In the 16S rDNA tree all *M. chelonae* isolates clustered with *M. saopaulense* CCUG 66554^T^. Moreover, it was not possible to discriminate *M. chelonae* ATCC 35752^T^ from the *M. franklinii* isolates as well isolates of genomospecies G1 and genomospecies G2. In the *hsp65* tree, isolates of genomospecies G1 clustered with *M. salmoniphilum* ATCC 13758^T^ and isolates D16Q19 and D16R27 did not cluster with *M. franklinii* DSM 45524^T^. Moreover, isolate D16Q15 did not cluster with *M. salmoniphilum* ATCC 13758^T^. In the 16S–23S ITS tree, isolate D16Q15 clustered with isolates of genomospecies G1 and not with *M. salmoniphilum* ATCC 13758^T^. Furthermore, isolates of genomospecies G3 were not grouped. In the *rpoB* and the concatenated trees all *M. chelonae* isolates clustered with *M. chelonae* ATCC 35752^T^, D16R27, and D16Q19 with *M. franklinii* DSM 45524^T^, and D16Q15 with *M. salmoniphilum* ATCC 13758^T^. Moreover, genomospecies G1, G2 and G3 formed clusters separated from all type strains (Figure 2). Therefore, *rpo*B and concatenated trees were in agreement with the isolates distribution obtained using ANI and GGD procedures.

## Discussion

The variability observed during the development of the INNO-LiPA® assay suggested the presence of different taxonomic groups among the CHII isolates. This genetic heterogeneity was already observed by Mijs et al. (2002). In the present study, we performed various whole genome sequence based analyses along with single gene sequencing and a biochemical characterization to characterize 26 INNO-LiPa cluster CHII isolates and included type strains of the established species belonging to the *M. chelonae*-*M. abscessus* complex.

Phenotypic analyses and drug susceptibility tests were not informative for distinguishing the taxonomic groups delineated by genomic analyses. Previous studies performed when the *M. chelonae-M. abscessus* complex comprised only two species, i.e., *M. chelonae* and *M. abscessus*, suggested that growth in the presence of 5% NaCl, the use of citrate as the sole carbon source and susceptibility to tobramycin were useful for distinguishing these two species (Yakrus et al., 2001). With the description of additional species, it became clear that phenotypic tests were not discriminative for species separation within this complex, as confirmed here and in other publications (Nogueira et al., 2015a,b).

Genomic analyses confirmed that the three subspecies within *M. abscessus* indeed represent a single species. *M. abscessus* subsp. *abscessus* ATCC 19977^T^, *M. abscessus* subsp. *bolletii* CCUG 50184^T^, and *M. abscessus* subsp. *massiliense* CCUG 48898^T^ showed a GGD value higher than the proposed cutoff (0.0266 to 0.0288 distance values); this result is in agreement with the recent data found by Tortoli and co-workers (Tortoli et al., 2016) when describing the subspecies within *M. abscessus*. However, the calculated dDDH percentages were higher than 70%, therefore within the value expected for a single species (Supplementary Table 6).

Analysis of the remaining type strains through the determination of ANIb, ANIm, TETRA, delta, GGD-dDDH values revealed that species delineation threshold values that are commonly used cannot consistently be applied to these closely related *Mycobacterium* species. This observation was further endorsed through the analysis of some of the cluster CHII isolates where e.g., ANI analyses demonstrated that some strains represented a single species while GGD and dDDH suggested they represented closely related yet distinct species. This was the case for 14 isolates that were grouped with *M. chelonae* ATCC 35752^T^ by ANI but not by GGD or dDDH, which suggested they represented a distinct species closely related to *M. chelonae*. In a similar manner, ANI and dDDH values assigned the isolate D16Q15 to *M. salmoniphilum* and the isolates D16Q19, D16R27 to *M. franklinii* while GGD values suggested they represented distinct species, closely related to *M. salmoniphilum* ATCC 13758^T^ and *M. franklinii* DSM 45524^T^, respectively (see Supplementary Tables 6, 8). In addition, the threshold level of 0.99 to discriminate species by means of their genomic TETRA values proved inadequate as the TETRA value of every pair of strains examined in the present study was consistently above 0.99, even in the case that other approaches, such as ANI, indicated that they were different species (see Supplementary Table 7). Similarly, delta values proved to be not discriminatory either (see Supplementary Table 9).

Finally, the genomic data also showed that the remaining nine isolates represent at least three novel species closely related to *M. salmoniphilum*. The genomic parameters for genomospecies G1 and G2 are consistent for grouping these isolates in separate species. For genomospecies G3 however, ANI values demonstrate that the isolates D16R12 and D16R18 represent a single species while GGD and dDDH data suggest they represent two species (0.0480 and 62.2%, respectively). When Whipps et al. proposed to revive the name *M. salmoniphilum* in 2007, a high variability among *M. salmoniphilum* isolates was observed (Whipps et al., 2007). Moreover, 16S rDNA and *hsp65* sequences of isolates of genomospecies G1, G2, and G3 showed a high similarity with the respective sequences of isolates recovered from fish, especially salmonids, in different geographic regions—Japan, Russia, Norway, Scotland, USA and Chile—and from tap water in the Netherlands (Whipps et al., 2007; van Ingen et al., 2010; Righetti et al., 2014) (data not shown). Together, these findings indicate that the *M. salmoniphilum* lineage comprises a broad group of closely related species that could represent a species complex in its own right.

Taken together, our results demonstrate the difficulties in assigning general cutoff for bacterial species separation using whole genome comparative techniques, thus stressing the utility in using more than one metric when comparing isolates.

In the present study we analyzed the variation among isolates of the INNO-LiPa cluster CHII using procedures that represent today's state-of-the-art in the analysis of WGS for taxonomic purposes. Our results showed that the current threshold values applied for WGS species delineation are not universally applicable, as exemplified by organisms of the *M. chelonae-M. abscessus* complex. Whole genome sequencing is still not routinely available for diagnostic purposes, making the analysis of few genes or informative genomic regions the standard procedure to identify difficult mycobacteria. However, recent advances in low-cost next-generation sequence technologies make it now possible to perform large-scale comparative studies. Individual and concatenated phylogenetic trees of taxonomically informative sequences were constructed to evaluate if they could accurately discriminate the species/groups established by ANI and GGD and be useful for the identification of these taxa in routine laboratories. Only the *rpoB* and the concatenated phylogenetic tree clearly showed the same taxonomic groups discriminated by ANI and GGD analyses (Figure 2), therefore, comparison of these sequences could accurately be used in the identification of members of the *M. chelonae-M. abscessus* complex until WGS could enter into the laboratory diagnostic routine.

## Author contributions

All authors contributed for drafting the work or revising it critically for important intellectual content, approved the version to be published and agreed to be accountable for all aspects of the work in ensuring that questions related to the accuracy or integrity of any part of the work are appropriately investigated and resolved. CN, MG, FP, PV, and SL contributed to the conception or design of the work. CN, LGPdA, MM, MG, LAD, EC, MC, JP, and AM contributed to the acquisition, analysis, or interpretation of data for the work.

## Funding

This study received financial support from Fundação de Amparo à Pesquisa do Estado de São Paulo (www.fapesp.br) (FAPESP) (grant 2011/18326-4). CN received a fellowship from FAPESP (2012/13763-0).

### Conflict of interest statement

The authors declare that the research was conducted in the absence of any commercial or financial relationships that could be construed as a potential conflict of interest.
