# Supplementary material for: Characterization of Mycobacterium chelonae-Like Strains by Comparative Genomics
Source: Front Microbiol. 2017 May 8;8:789. doi: 10.3389/fmicb.2017.00789 (PMC5420552; doi:10.3389/fmicb.2017.00789)
Supplement: Supplementary file 1 [file Table1.DOCX]

**Supplementary Table 1 -** Primers used for amplification and/or sequencing of 16S rDNA, *hsp65, rpoB* and 16S-23S internal transcribed sequence

| **Gene** | **Primers** | **Sequence 5´-3´** | **References** |
| --- | --- | --- | --- |
| 16S rRNA | 16S-27F# | AGAGTTTGATCCTGGCTCAG | (Harmsen et al., 2003) |
| 16S rRNA | 800F | ATTAGATACCCTGGTAG | (Adekambi and Drancourt, 2004) |
| 16S rRNA | 16S-907R# | CCGTCAATTCCTTTGAGTTT | (Harmsen et al., 2003) |
| 16S rRNA | 16R-1492# | TACGGCTACCTTGTTACGACTT | (Gomila et al., 2007) |
| *rpoB* | MYCOF1 | TCCGATGAGGTGCTGGCAGA | (Macheras et al., 2011) |
| *rpoB* | MYCOR2 | ACTTGATGGTCAACAGCTCC | (Macheras et al., 2011) |
| *hsp65* | hsp667F | GGCCAAGACAATTGCGTACG | (Selvaraju et al., 2005) |
| *hsp65* | hsp667R | GGAGCTGACCAGCAGGATG | (Selvaraju et al., 2005) |
| ITS | Sp1 | ACCTCCTTTCTAAGGAGCACC | (Roth et al., 2000) |
| ITS | 23S1R | CCCAAAGCCTATATATTCAGC | (Matsumoto et al., 2012) |

# Primers modified based on the 16S rDNA sequence of *M. abscessus* subsp. *abscessus* ATCC 19977

Adekambi, T., and Drancourt, M. (2004). Dissection of phylogenetic relationships among 19 rapidly growing *Mycobacterium* species by 16S rRNA, *hsp65*, *sodA*, *recA* and *rpoB* gene sequencing. *Int. J. Syst. Evol. Microbiol.* **54,** 2095-2105. doi: 10.1099/ijs.0.63094-0

Gomila, M., Ramirez, A., and Lalucat, J. (2007). Diversity of environmental *Mycobacterium* isolates from hemodialysis water as shown by a multigene sequencing approach. *Appl. Environ. Microbiol.* **73**:12**,** 3787-3797. doi: 10.1128/AEM.02934-06

Harmsen, D., Dostal, S., Roth, A., Niemann, S., Rothganger, J., Sammeth, M., et al. (2003). RIDOM: comprehensive and public sequence database for identification of *Mycobacterium* species. *BMC Infect. Dis.* **3**: 26. doi: 10.1186/1471-2334-3-26

Macheras, E., Roux, A.L., Bastian, S., Leao, S.C., Palaci, M., Sivadon-Tardy, V., et al. (2011). Multilocus sequence analysis and *rpoB* sequencing of *Mycobacterium abscessus* (sensu lato) strains. *J. Clin. Microbiol.* **49**:2**,** 491-499. doi: 10.1128/JCM.01274-10

Matsumoto, C.K., Chimara, E., Ramos, J.P., Campos, C.E., Caldas, P.C., Lima, K.V., et al. (2012). Rapid tests for the detection of the *Mycobacterium* *abscessus* subsp. *bolletii* strain responsible for an epidemic of surgical-site infections in Brazil. *Mem. Inst. Oswaldo Cruz* **107**:8**,** 969-977. doi: 10.1590/S0074-02762012000800002

Roth, A., Reischl, U., Streubel, A., Naumann, L., Kroppenstedt, R.M., Habicht, M., et al. (2000). Novel diagnostic algorithm for identification of mycobacteria using genus-specific amplification of the 16S-23S rRNA gene spacer and restriction endonucleases. *J. Clin. Microbiol.* **38**:3**,** 1094-1104.

Selvaraju, S.B., Khan, I.U., and Yadav, J.S. (2005). A new method for species identification and differentiation of *Mycobacterium chelonae* complex based on amplified *hsp65* restriction analysis (AHSPRA). *Mol. Cell Probes* **19**:2**,** 93-99. doi: 10.1016/j.mcp.2004.09.007
