# Supplementary material for: Characterization of Mycobacterium chelonae-Like Strains by Comparative Genomics
Source: Front Microbiol. 2017 May 8;8:789. doi: 10.3389/fmicb.2017.00789 (PMC5420552; doi:10.3389/fmicb.2017.00789)
Supplement: Supplementary file 2 [file Table2.DOCX]

**Supplementary Table 2 -** *Mycobacterium* species, strains and GenBank accession numbers used for construction of single-gene and concatenated trees

| **Gene** | ***Mycobacterium* species** | **Strain** | **Accession number** |
| --- | --- | --- | --- |
| 16S rDNA | *M. abscessus* subsp. *abscessus* | CIP 104536^T^ | AY457071 |
|  | *M. abscessus* subsp. *bolletii* | CIP 108541^T^ | AY859681 |
|  | *M. abscessus* subsp. *massiliense* | CCUG 48898^T^ | NR_043002 |
|  | *M. chelonae* | CIP 104535^T^ | AY457072 |
|  | *M. immunogenum* | DSM 45595^T^ | HE654001 |
|  | *M. salmoniphilum* | ATCC 13758^T^ | NR_043989 |
|  | *M. franklinii* | DSM 45524^T^ | HQ153090 |
|  | *M. saopaulense* | CCUG 66554^T^ | KM973037 |
|  | *M. tuberculosis* | H37Rv | NC_000962_3 |
|  | *M. smegmatis* | mc^2^155 | NC_008596 |
| *hsp65* | *M. abscessus* subsp. *abscessus* | CIP 104536^T^ | AY458075 |
|  | *M. abscessus* subsp. *bolletii* | CIP 108541^T^ | AY859675 |
|  | *M. abscessus* subsp. *massiliense* | CCUG 48898^T^ | AY596465 |
|  | *M. chelonae* | CIP 104535^T^ | AY458074 |
|  | *M. immunogenum* | CIP 106684^T^ | AY458081 |
|  | *M. salmoniphilum* | ATCC 13758^T^ | DQ866777 |
|  | *M. franklinii* | DSM 45524^T^ | KM392059 |
|  | *M. saopaulense* | CCUG 66554^T^ | KM973026 |
|  | *M. tuberculosis* | H37Rv | NC_000962_3 |
|  | *M. smegmatis* | mc^2^155 | NC_008596 |
| *rpoB* | *M. abscessus* subsp. *abscessus* | CIP 104536^T^ | AY147164 |
|  | *M. abscessus* subsp. *bolletii* | CIP 108541^T^ | AY859692 |
|  | *M. abscessus* subsp. *massiliense* | CCUG 48898^T^ | AY593981.2 |
|  | *M. chelonae* | CIP 104535^T^ | AY147163 |
|  | *M. immunogenum* | CIP 106684^T^ | AY262739 |
|  | *M. salmoniphilum* | ATCC 13758^T^ | KM392058 |
|  | *M. franklinii* | DSM 45524^T^ | KM392056 |
|  | *M. saopaulense* | CCUG 66554^T^ | KM973029 |
|  | *M. tuberculosis* | H37Rv | NC_000962_3 |
|  | *M. smegmatis* | mc^2^155 | NC_008596 |
| ITS | *M. abscessus* subsp. *abscessus* | CIP 104536^T^ | AY593976 |
|  | *M. abscessus* subsp. *bolletii* | CCUG 50184^T^ | GU143888.2 |
|  | *M. abscessus* subsp. *massiliense* | CCUG 48898^T^ | AY593978.1 |
|  | *M. chelonae* | ATCC 35752^T^ | AY498739 |
|  | *M. immunogenum* | CIP 106684^T^ | AY593977 |
|  | *M. salmoniphilum* | ATCC 13758^T^ | DQ866768 |
|  | *M. franklinii* | DSM 45524^T^ | HQ153093 |
|  | *M. saopaulense* | CCUG 66554^T^ | KM973034 |
|  | *M. tuberculosis* | ATCC 27294^T^ | L15623 |
|  | *M. smegmatis* | mc^2^155 | NC_008596 |
