# Supplementary material for: Characterization of Mycobacterium chelonae-Like Strains by Comparative Genomics
Source: Front Microbiol. 2017 May 8;8:789. doi: 10.3389/fmicb.2017.00789 (PMC5420552; doi:10.3389/fmicb.2017.00789)
Supplement: Supplementary file 5 [file Table5.DOCX]

**Supplementary Table 5 -** Illumina Miseq assembly details for the isolates and type strains of *M. chelonae-M. abscessus* group

| **Isolates and type strains** | **Bases assembled** | **Number of scaffolds** | **N50 scaffolds size (bp)** | **Estimated %GC content** |
| --- | --- | --- | --- | --- |
| 96-1705 | 4,998,263 | 29 | 270,676 | 63.98 |
| 96-1717 | 5,088,556 | 23 | 533,807 | 63.79 |
| D16Q24 | 5,457,716 | 50 | 336,599 | 63.82 |
| 96-1720 | 4,989,632 | 27 | 281,017 | 63.89 |
| 96-1724 | 5,167,153 | 34 | 433,022 | 63.90 |
| 96-1728 | 4,940,199 | 22 | 569,132 | 64.07 |
| D16R2 | 5,153,513 | 23 | 350,269 | 63.85 |
| D16R3 | 5,201,663 | 21 | 350,642 | 63.96 |
| D16R7 | 5,212,684 | 20 | 500,734 | 63.89 |
| D16R9 | 5,126,063 | 23 | 362,638 | 63.88 |
| D16R10 | 5,133,965 | 48 | 187,245 | 63.90 |
| D16R14 | 5,123,766 | 21 | 575,060 | 63.91 |
| D16R19 | 4,989,084 | 24 | 306,045 | 63.95 |
| D16R20 | 5,182,981 | 41 | 250,323 | 63.92 |
| 96-892 | 4,931,770 | 37 | 458,987 | 64.13 |
| D16Q13 | 4,903,783 | 23 | 579,383 | 64.18 |
| D16Q16 | 5,017,561 | 23 | 370,841 | 64.19 |
| D16Q14 | 5,171,220 | 65 | 198,484 | 63.77 |
| D16Q20 | 4,858,082 | 73 | 134,797 | 63.93 |
| D16R24 | 5,218,400 | 53 | 219,363 | 63.81 |
| D17A2 | 5,201,188 | 56 | 219,356 | 63.76 |
| D16Q15 | 4,939,516 | 15 | 682,599 | 63.63 |
| D16R12 | 4,808,095 | 27 | 294,619 | 64.08 |
| D16R18 | 4,991,856 | 82 | 110,426 | 63.89 |
| D16Q19 | 5,112,070 | 38 | 477,240 | 63.93 |
| D16R27 | 5,120,456 | 19 | 588,891 | 64.00 |
| *M. abscessus* subsp. *boletii*  CCUG 50184^T^ | 5,044,397 | 21 | 443,298 | 63.90 |
| *M. immunogenum*  ATCC 700505^T^ | 5,548,818 | 19 | 406,873 | 64.04 |
| *M. salmoniphilum*  ATCC 13758^T^ | 4,768,278 | 25 | 250,904 | 64.15 |
| *M. franklinii*  DSM 45524^T^ | 5,437,336 | 29 | 325,961 | 64.14 |
