# Supplementary material for: Characterization of Mycobacterium chelonae-Like Strains by Comparative Genomics
Source: Front Microbiol. 2017 May 8;8:789. doi: 10.3389/fmicb.2017.00789 (PMC5420552; doi:10.3389/fmicb.2017.00789)
Supplement: Supplementary file 9 [file Table9.PDF]

Supplementary Table 9. Delta values obtained with the 26 isolates and the *M. chelonae-M. abscessus* group type strains

δ*-differences

Sample size: 50 kb

closely similar | moderately similar | weakly similar | distantly similar | distant | very distant

10 20 30 40 50 60 70 80 90 100 110 120 130 140 150 160 170 180 190 200 210 220 230 240 250 260 270 280 290 300

	Seq AA	Seq AB	Seq AC	Seq AD	Seq AE	Seq AF	Seq AG	Seq AH	Seq AI	Seq AJ	Seq AK	Seq AL	Seq AM	Seq AN	Seq AO	Seq AP	Seq AQ	Seq AR	Seq AS	Seq AT	Seq AU	Seq AV	Seq AW	Seq AX	Seq AY	Seq AZ	Seq BA	Seq BB	Seq BC	Seq BD	Seq BE	Seq BF	Seq BG	Seq BH
	101	104	101	98	100	102	100	103	99	109	103	104	104	102	103	102	100	104	102	109	102	111	99	95	99	98	103	100	97	96	100	104	104	93
SeqAA	**24**	**23**	**23**	**25**	**26**	**27**	**26**	**26**	**25**	**26**	**26**	**27**	**27**	**26**	**25**	**26**	**25**	**25**	**25**	**26**	**25**	**25**	**26**	**25**	**26**	**26**	**26**	**27**	**26**	**27**	**25**	**27**	**27**	**28**
SeqAB		**23**	**22**	**25**	**26**	**27**	**26**	**26**	**25**	**26**	**26**	**27**	**27**	**26**	**25**	**26**	**25**	**25**	**25**	**26**	**25**	**25**	**26**	**25**	**26**	**26**	**26**	**27**	**26**	**27**	**25**	**27**	**27**	**28**
SeqAC			**22**	**24**	**25**	**26**	**25**	**25**	**24**	**26**	**25**	**26**	**26**	**25**	**25**	**25**	**25**	**24**	**25**	**25**	**25**	**24**	**25**	**25**	**25**	**26**	**26**	**26**	**26**	**26**	**25**	**26**	**26**	**27**
SeqAD				**22**	**22**	**24**	**23**	**23**	**22**	**24**	**23**	**24**	**24**	**23**	**22**	**23**	**22**	**22**	**23**	**23**	**23**	**26**	**23**	**21**	**22**	**22**	**23**	**23**	**22**	**22**	**21**	**23**	**23**	**22**
SeqAE					**23**	**24**	**23**	**23**	**23**	**24**	**23**	**24**	**24**	**24**	**23**	**23**	**23**	**23**	**24**	**24**	**24**	**26**	**23**	**22**	**22**	**23**	**23**	**23**	**23**	**23**	**22**	**24**	**24**	**23**
SeqAF						**26**	**25**	**25**	**24**	**26**	**25**	**26**	**26**	**25**	**25**	**25**	**24**	**24**	**25**	**26**	**25**	**28**	**25**	**24**	**24**	**24**	**25**	**25**	**24**	**24**	**24**	**25**	**25**	**25**
SeqAG							**24**	**24**	**23**	**24**	**24**	**25**	**25**	**24**	**23**	**24**	**23**	**23**	**24**	**25**	**24**	**27**	**24**	**23**	**23**	**23**	**24**	**24**	**23**	**23**	**23**	**24**	**24**	**24**
SeqAH								**24**	**23**	**25**	**24**	**25**	**25**	**24**	**23**	**24**	**23**	**23**	**24**	**25**	**24**	**27**	**24**	**23**	**23**	**23**	**24**	**24**	**23**	**23**	**23**	**24**	**24**	**24**
SeqAI									**22**	**24**	**23**	**24**	**24**	**23**	**23**	**23**	**22**	**22**	**23**	**24**	**24**	**26**	**23**	**22**	**22**	**22**	**23**	**23**	**23**	**22**	**22**	**23**	**24**	**23**
SeqAJ										**25**	**25**	**25**	**25**	**25**	**24**	**25**	**24**	**24**	**25**	**25**	**25**	**27**	**24**	**24**	**24**	**24**	**25**	**25**	**24**	**24**	**24**	**25**	**25**	**25**
SeqAK											**24**	**25**	**25**	**24**	**24**	**24**	**23**	**23**	**24**	**25**	**24**	**27**	**24**	**23**	**23**	**23**	**24**	**24**	**23**	**23**	**23**	**24**	**24**	**24**
SeqAL												**26**	**25**	**25**	**24**	**25**	**24**	**24**	**25**	**26**	**25**	**28**	**25**	**24**	**24**	**24**	**25**	**25**	**24**	**24**	**24**	**25**	**25**	**24**
SeqAM													**26**	**25**	**24**	**25**	**24**	**24**	**25**	**25**	**25**	**28**	**25**	**23**	**24**	**24**	**25**	**25**	**24**	**24**	**23**	**25**	**25**	**24**
SeqAN														**25**	**24**	**24**	**23**	**23**	**24**	**25**	**25**	**27**	**24**	**23**	**23**	**24**	**24**	**24**	**24**	**24**	**23**	**25**	**25**	**24**
SeqAO															**23**	**24**	**23**	**23**	**24**	**24**	**24**	**26**	**23**	**22**	**23**	**23**	**24**	**24**	**23**	**23**	**22**	**24**	**24**	**24**
SeqAP																**24**	**23**	**23**	**24**	**25**	**24**	**27**	**24**	**23**	**23**	**23**	**24**	**24**	**23**	**23**	**23**	**24**	**24**	**23**
SeqAQ																	**22**	**22**	**23**	**24**	**24**	**26**	**23**	**22**	**22**	**23**	**23**	**23**	**23**	**22**	**22**	**23**	**24**	**23**
SeqAR																		**22**	**23**	**24**	**24**	**26**	**23**	**22**	**22**	**23**	**23**	**23**	**23**	**23**	**22**	**23**	**24**	**23**
SeqAS																			**24**	**24**	**24**	**26**	**24**	**23**	**23**	**24**	**24**	**24**	**24**	**23**	**23**	**25**	**25**	**24**
SeqAT																				**25**	**24**	**27**	**25**	**23**	**24**	**24**	**25**	**25**	**24**	**24**	**24**	**25**	**25**	**24**
SeqAU																					**24**	**26**	**24**	**23**	**23**	**24**	**25**	**24**	**24**	**24**	**23**	**25**	**25**	**24**
SeqAV																						**27**	**27**	**27**	**27**	**27**	**27**	**28**	**27**	**27**	**26**	**28**	**28**	**28**
SeqAW																							**23**	**22**	**22**	**23**	**23**	**23**	**23**	**23**	**22**	**24**	**24**	**24**
SeqAX																								**20**	**21**	**21**	**22**	**22**	**22**	**21**	**21**	**23**	**23**	**21**
SeqAY																									**21**	**22**	**23**	**22**	**22**	**22**	**21**	**23**	**23**	**22**
SeqAZ																										**22**	**23**	**23**	**22**	**22**	**22**	**23**	**23**	**23**
SeqBA																											**24**	**24**	**23**	**23**	**23**	**24**	**24**	**24**
SeqBB																												**24**	**23**	**23**	**22**	**24**	**24**	**23**
SeqBC																													**23**	**22**	**22**	**23**	**24**	**23**
SeqBD																														**22**	**22**	**23**	**23**	**22**
SeqBE																															**21**	**23**	**23**	**22**
SeqBF																																**24**	**24**	**24**
SeqBG																																	**25**	**24**
SeqBH																																		**21**

SeqAA *M. abscessus* subsp. *abscessus* ATCC19977
SeqAB *M. abscessus* subsp. *massiliense* CCUG48898
SeqAC *M. abscessus* subsp. *bolletii* CCUG50184
SeqAD *M. chelonae* ATCC35752
SeqAE 961705
SeqAF 961717
SeqAG 961720
SeqAH 961724
SeqAI 961728
SeqAJ D16Q24
SeqAK D16R2
SeqAL D16R3
SeqAM D16R7
SeqAN D16R9
SeqAO D16R10
SeqAP D16R14
SeqAQ D16R19
SeqAR D16R20
SeqAS D16R27
SeqAT *M. franklinii* DSM45524
SeqAU D16Q19
SeqAV *M. immunogenum* ATCC700505
SeqAW D16Q15
SeqAX *M. salmoniphilum* ATCC13758
SeqAY 96892
SeqAZ D16Q13
SeqBA D16Q14
SeqBB D16Q16
SeqBC D16Q20
SeqBD D16R12
SeqBE D16R18
SeqBF D16R24
SeqBG D17A2
SeqBH *M. saopaulense* CCUG66554
