# Supplementary figures and images for: Characterization of Mycobacterium chelonae-Like Strains by Comparative Genomics
Source: Front Microbiol. 2017 May 8;8:789. doi: 10.3389/fmicb.2017.00789 (PMC5420552; doi:10.3389/fmicb.2017.00789)

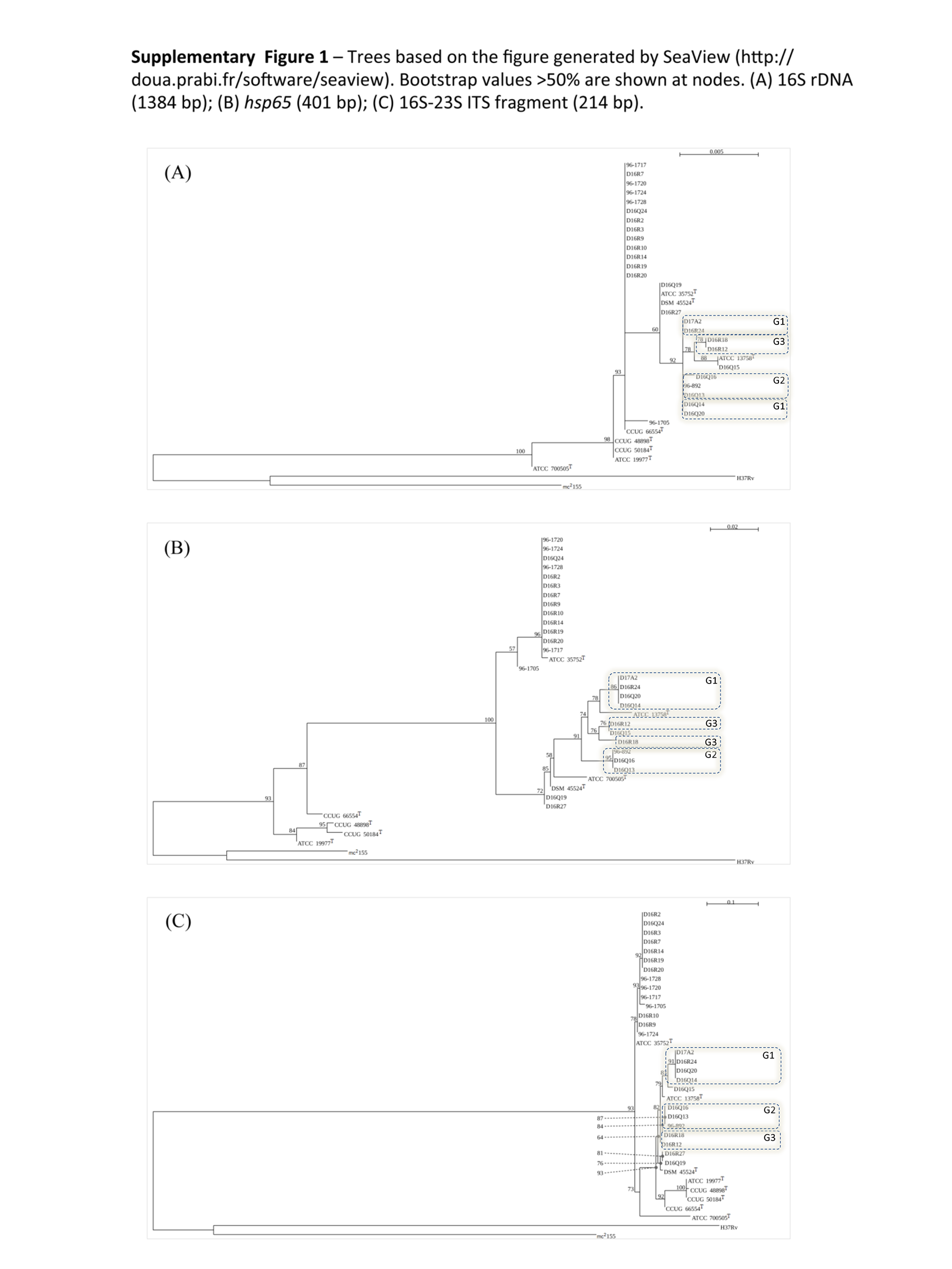

Supplement: Supplementary file 10 [file Image1.TIF]
